# Gene Dosage Effects of the Imprinted Delta-Like Homologue 1 (*Dlk1*/*Pref1*) in Development: Implications for the Evolution of Imprinting

**DOI:** 10.1371/journal.pgen.1000392

**Published:** 2009-02-27

**Authors:** Simao Teixeira da Rocha, Marika Charalambous, Shau-Ping Lin, Isabel Gutteridge, Yoko Ito, Dionne Gray, Wendy Dean, Anne C. Ferguson-Smith

**Affiliations:** 1 Department of Physiology, Development and Neuroscience, University of Cambridge, Cambridge, United Kingdom; 2 Institute of Biotechnology, College of Bioresources and Agriculture, National Taiwan University, Taipei, Taiwan; 3 Developmental Genetics and Imprinting Laboratory, The Babraham Institute, Cambridge, United Kingdom; The Babraham Institute, United Kingdom

## Abstract

Genomic imprinting is a normal process that causes genes to be expressed according to parental origin. The selective advantage conferred by imprinting is not understood but is hypothesised to act on dosage-critical genes. Here, we report a unique model in which the consequences of a single, double, and triple dosage of the imprinted *Dlk1*/*Pref1*, normally repressed on the maternally inherited chromosome, can be assessed in the growing embryo. BAC-transgenic mice were generated that over-express *Dlk1* from endogenous regulators at all sites of embryonic activity. Triple dosage causes lethality associated with major organ abnormalities. Embryos expressing a double dose of *Dlk1*, recapitulating loss of imprinting, are growth enhanced but fail to thrive in early life, despite the early growth advantage. Thus, any benefit conferred by increased embryonic size is offset by postnatal lethality. We propose a negative correlation between gene dosage and survival that fixes an upper limit on growth promotion by *Dlk1*, and we hypothesize that trade-off between growth and lethality might have driven imprinting at this locus.

## Author Summary

Genomic imprinting, the process that causes genes to be expressed from one of the two chromosome homologues according to parental origin, is likely to act on genes whose dosage is important for their correct function. To test this, we compared the phenotype of transgenic mice expressing a double and triple dose of the imprinted gene *Dlk1/Pref1* with animals expressing the normal single dose expressed from the paternally inherited chromosome. Our results showed that a triple dose causes severe developmental abnormalities and death before or at birth. Embryos expressing a double dose, recapitulating absence of imprinting, are bigger at birth but then around one-third of them died within the first three days of life. Those that survived had poor early growth performance in the first week of life becoming small and remaining small, thus offsetting any benefit conferred by being born bigger. Therefore, imprinted levels of *Dlk1/Pref1* represent the optimal balance of growth versus lethality. These findings lead to speculation about the evolutionary pressures acting to establish and maintain imprinting at this locus.

## Introduction

Genomic imprinting, the process causing genes to be differentially expressed according to their parental origin acts on a subset of developmentally regulated genes [reviewed in 1]. Since imprinting results in functional haploidy of target genes, the evolution of such a mechanism must have conferred a significant advantage to its recipients to offset the cost. In addition, the maintenance of this method of gene dosage regulation must confer a continuing selective benefit to the individual. By creating genetic models that mimic the loss of imprinting of genes regulated in this way, we can study phenotype and consider what selective pressures act to maintain gene dosage, and begin to examine what may have driven the acquisition of mono-allelic expression.

*Dlk1*, *Delta-like homologue 1* also known as *Preadipocyte factor 1* (*Pref1*), on mouse chromosome 12 is part of an imprinted gene cluster [2],[3]. It encodes a transmembrane glycoprotein that possesses six epidermal growth factor-like motifs in the extracellular domain similar to those present in the *Delta/Notch/Serrate* family of signalling molecules. In contrast to other NOTCH ligands, DLK1 does not have the *Delta:Serrate:Lin-12 (DSL)* domain believed to mediate the interaction and activation of the NOTCH receptor [4]. Nevertheless, DLK1 can interact with NOTCH through specific *EGF*-like repeats and can act as a *Notch* antagonist, both in culture and *in vivo*, binding to the receptor without activating it [5],[6],[7]. *In vitro*, *Dlk1* maintains precursor cell populations and inhibits differentiation [4],[8],[9]. *Dlk1* is expressed at high levels in a wide range of embryonic tissues [10],[11] however its developmental functions *in vivo* are largely unknown.

In more ancestral vertebrates such as the fish *Oryzias latipes* and *Fugu rubripes*, *Dlk1*, and its neighbour *Dio3* which encodes a negative regulator of thyroid hormone metabolism, are only 10–15 Kb apart. In eutherian mammals, the genes have become separated by the insertion of sequences including a retrotransposon-like gene (*Rtl1*) and several non-coding RNAs, including a large microRNA cluster, and have acquired imprinting [12]. *Dlk1*, *Rtl1* and *Dio3* are expressed from the paternally inherited chromosome and the non-coding RNAs from the maternally-inherited chromosome [13].

Imprinting on chromosome 12 is controlled by an intergenic differentially methylated region (IG-DMR) that is methylated on the paternally inherited chromosome [14],[15]. The unmethylated maternal IG-DMR is necessary to repress protein-coding transcription and to activate the non-coding RNAs [14]. In terms of gene expression, the paternal chromosome 12 most likely resembles the ancestral (pre-imprinted) state as *Dlk1* and *Dio3* are expressed and the non-coding RNAs are silenced [12]. Furthermore, paternal deletion of the methylated IG-DMR has no effect on transcription [14].

Alteration of imprinting at chromosome 12 has considerable consequences for embryonic fitness. Uniparental disomy mice with two paternal copies and no maternal copy (PatDi(12)/PatDp(dist12)) or mice with two maternal copies and no paternal copy (MatDi(12)/MatDp(dist12)) of chromosome 12, are lethal and show distinct phenotypes. These include growth abnormalities and developmental defects in muscle, cartilage/bone and placenta [16],[17],[18]. Animals die prenatally commencing at E16. The embryonic uniparental disomic phenotypes can be ascribed to the *Dlk1-Dio3* imprinted cluster because embryos with maternal deletion of the IG-DMR (ΔIG-DMR^MAT^) recapitulate the transcriptional profile and embryonic mutant phenotypes of the PatDi(12)/PatDp(dist12) conceptuses [14],[19].

Since loss of imprinting at chromosome 12 is lethal, there are clear advantages in maintaining the imprinted state. However since in PatDi(12)/PatDp(dist12) and ΔIG-DMR^MAT^ conceptuses multiple paternally expressed coding genes are up-regulated, and multiple maternally expressed non-coding genes are repressed, we are unable to determine if selection is acting to maintain the mono-allelic expression of one or all protein coding genes or to maintain expression of the non-coding RNAs. As *Dlk1* is an ancestral gene at this imprinted locus and it is expressed at high levels in tissues where uniparental disomy conceptuses have the most pronounced abnormalities [10], we consider *Dlk1* as a strong candidate for the pathological defects associated with the *Dlk1-Dio3* region. Therefore in this study, we specifically ask if imprinting might be maintained to prevent overdose of *Dlk1* and moreover, if selection to control the dosage of this gene might have driven the evolution of imprinting of the chromosome 12 cluster.

Manipulating imprinted gene dosage *in vivo* provides a powerful tool to study imprinted gene function and evolution. Here we describe a *Dlk1* over-expression model in which the gene is driven by its own endogenous regulatory sequences. We generate mouse lines harbouring bacterial artificial chromosome (BAC) transgenes that encompass the entire unmanipulated *Dlk1* gene with different lengths of flanking sequence. Of these, transgenic *Dlk1* expression at sites where the endogenous gene is expressed, was achieved from a 70 kb BAC transgene in three independent lines and the outcome was the same for all three lines. This allowed us to compare the phenotypes of mice expressing a double (transgene hemizygotes) or triple (transgene homozygotes) dose of *Dlk1* with normal animals expressing a single imprinted dose. This transgenic system of regulated *Dlk1* over-dose allowed us to explore the developmental and physiological functions of this gene in comparison with other models, and infer evolutionary scenarios for *Dlk1* imprinting.

## Results

### Construction of a Transgenic System for the Alteration of *Dlk1/Pref1* Dosage

**Figure 1:**
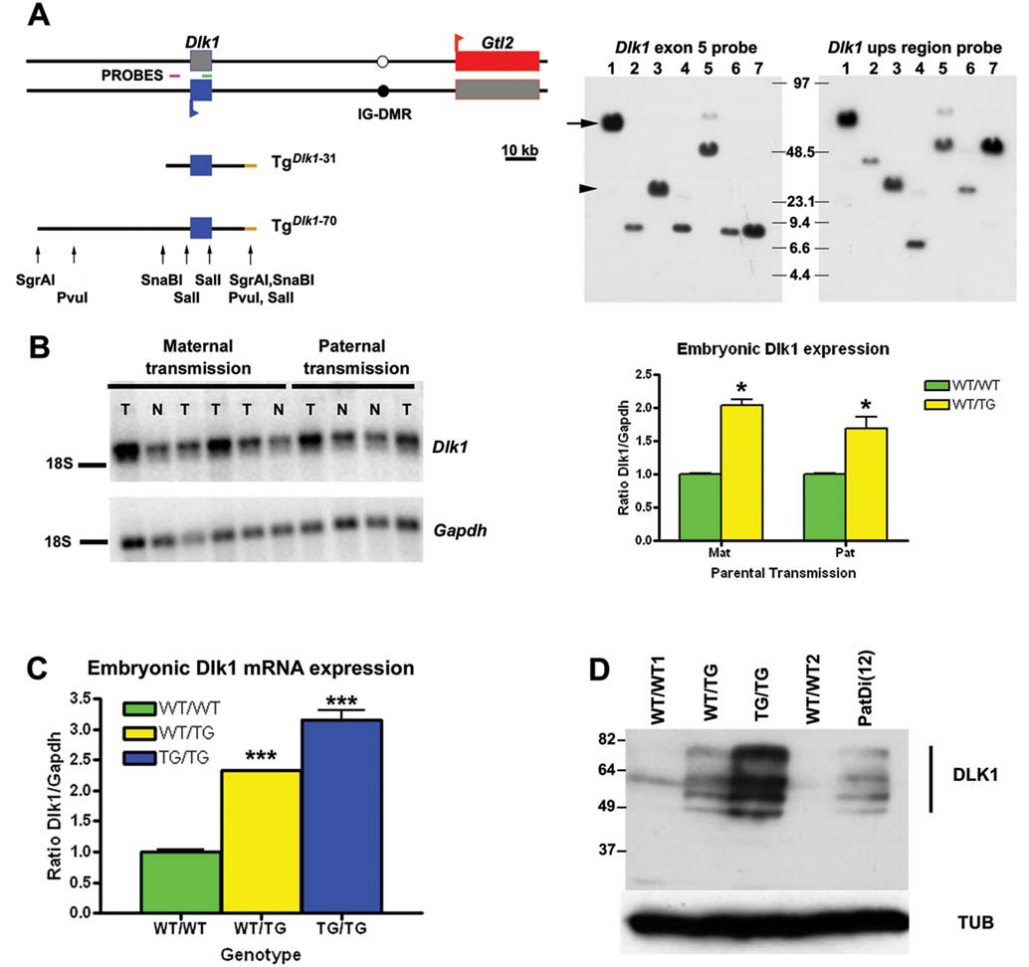
Generation of *Dlk1* transgenic mice. A. Two different BAC transgenes containing the *Dlk1* gene. The left panel displays a schematic representation of the two BAC transgenes containing *Dlk1* presented in this study: Tg*^Dlk1^* ^-31^ and Tg*^Dlk1^* ^-70^. Tg*^Dlk1^* ^-31^ did not express the transgene and was not further analysed. Red box - maternally expressed gene *Gtl2*; blue box – paternally expressed gene *Dlk1*; white circle – unmethylated IG-DMR; black circle – methylated IG-DMR; brown line – vector sequence; Probes: violet line – *Dlk1* upstream region probe, green line – *Dlk1* exon 5 probe. The right panel contains representative Southern hybridizations of the restriction enzyme fragments of the BAC 153O05 clone from where Tg*^Dlk1^* ^-31^ (arrowhead) and Tg*^Dlk1^* ^-70^ (arrow) were obtained by restriction digestion; the two probes were used to confirm the presence of appropriate restriction fragments containing the *Dlk1* gene and upstream sequences within the transgene. Lane 1: *Sgr*AI; Lane 2: *Sgr*AI+*Sal*I; Lane 3: *Sna*BI; Lane 4: *Sna*BI+*Sal*I; Lane 5: *Pvu*I; Lane 6: *Pvu*I+*Sal*I; Lane 7: *Sal*I. B. Expression of *Dlk1* in transgene hemizygotes is independent of parental inheritance of the transgene. Quantitative Northern Blot showing that *Dlk1* is over-expressed upon both maternal and paternal transmission of the Tg*^Dlk1^* ^-70^ transgene in E16 70C fetuses. The graph represents mean normalized *Dlk1* expression±SEM (ratio: *Dlk1/Gapdh*) from WT/TG and WT/WT littermates upon maternal or paternal transmission of the transgene. Significant differences between the WT/TG and WT/WT littermates are indicated by asterisks (p-value<0.05, unpaired Student's *t* test); Abbreviations: N – WT/WT fetus; T – WT/TG fetus; Mat – maternal transmission; Pat – paternal transmission. C. Double and triple dosage of *Dlk1* in hemizygous and homozygous Tg*^Dlk1^* ^-70^ transgenic mice. The graph on the left represents normalized *Dlk1* expression from E16 70B WT/WT, WT/TG and TG/TG fetuses from heterozygous intercrosses obtained by Northern Blot analysis (as in B). Significant differences between the WT/TG and WT/WT and between TG/TG and WT/WT are indicated by asterisks (p-value<0.01, unpaired Student's *t* test; n≥4 for all genotypes). D. Comparative DLK1 protein levels in transgenic and PatDi(12) E16 70B fetuses and control littermates were evaluated by Western Blot (right panel). Antibodies used were anti-DLK1 and anti α-TUBULIN (TUB) as a normalization control. Comparable protein isoforms are evident between WT/WT and the transgenic animals on longer exposure.

BAC transgenes were generated that encompass the entire *Dlk1* gene and endogenous flanking sequences but without other genes in the cluster. Transgenic mice were created by pronuclear injection of two BAC transgenes differing in the amounts of flanking regulatory sequences (Figure 1A): Tg*^Dlk1^* ^-31^ starts 8 kb upstream of the *Dlk1* gene and ends approximately 18 kb downstream of the *Dlk1* transcriptional start site. The Tg*^Dlk1^* ^-70^ transgene shares its 3′ end with the Tg*^Dlk1^* ^-31^ transgene but contains 49.4 kb of sequence upstream of *Dlk1* (Figure 1A). The four independent lines containing the Tg*^Dlk1^* ^-31^ transgene failed to express *Dlk1* and no *Dlk1*-associated phenotypes were observed (data not shown) regardless of transgene copy number (Figure S1A). These mice were not analysed further. In contrast, the Tg*^Dlk1^* ^-70^ transgenic animals successfully expressed *Dlk1* (Figure 1B and C and Table S1), and were subjected to further analysis.

The four independent Tg*^Dlk1^* ^-70^ lines of mice, hitherto referred to as 70A, 70B, 70C and 70D were maintained as hemizygotes (referred to as WT/TG) and bred to C57BL/6 for more than ten generations to ensure stability of copy number and phenotype. Copy number was stable from the third generation onwards and estimated to be 4–5 (70A), 5–6 (70B), 7 (70C) and 1 (70D) (Figure S1 and Table S1). Once stable lines were established, analysis of the overall level of *Dlk1* expression was performed by Northern Blotting for the different lines.

In the single copy 70D line, no transgene-derived *Dlk1* expression was observed and no phenotypic consequences were noted (data not shown). This line was not analysed further.

Importantly, for the 70A, 70B and 70C lines, WT/TG E16 embryos expressed approximately twice as much *Dlk1* as their normal littermates regardless of copy number. Representative quantitative Northern blot expression data is illustrated in Figure 1B and 1C for 70C and 70B families. Detailed expression data (both from Northern blots and RT-qPCR) are shown independently for the three lines in Figure S2B and STable S1). This expression analysis using two independent methods clearly shows that WT/TG E16 and E18 embryos for the three lines express approximately twice as much *Dlk1*.

Interestingly, expression of the transgene occurred independent of the parental-origin of the transgene in the three independent lines (Figure 1B; Table S1). Absence of transgene imprinting was confirmed using a single nucleotide polymorphism (SNP) located in the 3′UTR of *Dlk1* (Lin et al., 2003) allowing the endogenous gene to be distinguished from the transgene (Figure S2A; Table S1).

As the transgene is not imprinted, homozygous transgenic mice (TG/TG) were generated to further increase the dosage of *Dlk1* in the three transgene expressing lines. As illustrated in Figure 1C for 70B and shown also for 70A and 70C (Figure S2 and Table S1) levels of *Dlk1* in the E16–E18 TG/TG embryos were approximately 3-fold higher than normal littermates.

**Figure 2:**
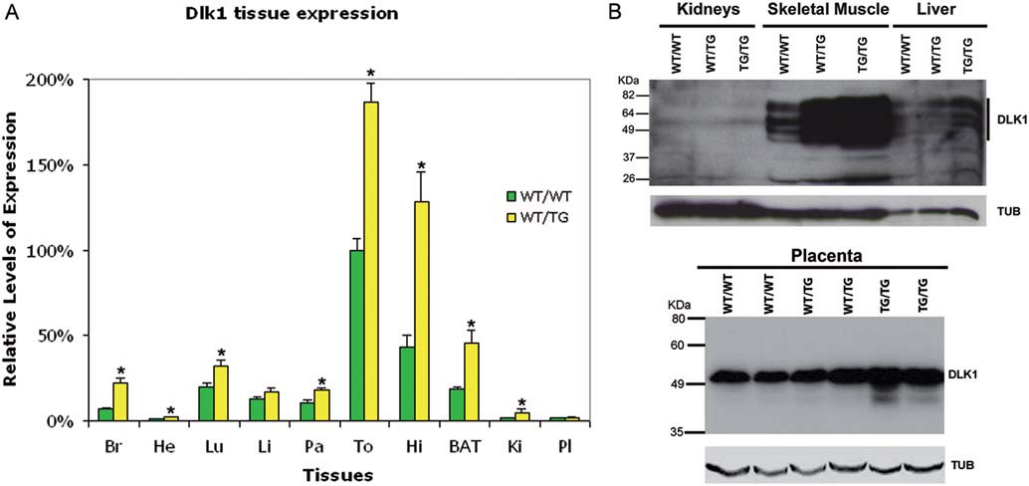
Tg*^Dlk1^* ^-70^ transgene recapitulates the expression of the endogenous locus in embryonic tissues but not in the placenta. A. *Dlk1* tissue expression analysis by TaqMan RT-qPCR shown for E18. Graphic representation of relative mean *Dlk1* expression in WT/WT and WT/TG tissues normalized to 100% E18 WT/WT tongue expression±SEM (n≥5). Data is shown for the 70B family. Significant differences between WT/TG and WT/WT for each tissue at each embryonic stage are indicated by asterisks on top of the WT/TG bar (p-value<0.05; unpaired Student's *t* test). Fold differences between wild-type and transgenic conceptuses are: Br: 3.21×±0.37 of *Dlk1* WT/WT expression; He: 2.55×±0.35; Lu: 1.61×±0.20, Li: 1.31×±0.21 NS; Pa: 1.65×±0.14; To: 1.87×±0.11, He: 2.99×±0.4, BAT: 2.41×±0.44; and Ki: 2.49×±0.21; Pla: 1.09×±0.15 Abbreviations: Br – brain; He – heart; Lu – lungs; Li – liver; Pa – pancreas; To – tongue; Hi – hindlimbs; BAT – Brown adipose tissue; Kid – kidneys; Pla – placenta. B. DLK1 protein levels in transgenic and wild-type tissues. Western blot analysis of DLK1 protein levels in E18 70B transgenic and WT/WT liver, muscle and kidney (negative control) and E16 placenta. For placenta, values normalised against tubulin are as follows: WT/WT = 1.00±0.017; WT/TG = 1.183±0.051 and TG/TG = 1.533^*^±0.073 (^*^for TG/TG p = 0.0417; paired non-parametric Freidman test). Antibodies used were anti-DLK1(DLK1) and anti α-TUBULIN (TUB) as a normalization control.

*Dlk1* regulation is complex involving alternative splicing and extensive post-translational modifications, therefore we compared DLK1 protein isoforms between the genotypes (for the 70B family). DLK1 protein levels in the different genotypes are consistent with the *Dlk1* mRNA expression and we saw no change in isoform preference (Figure 1D; Figure 2B). Furthermore, DLK1 protein levels in WT/TG embryos are comparable to that of PatDi(12) embryos, and are further increased in TG/TG conceptuses.

### Tg*^Dlk1^* ^-70^ Transgene Recapitulates the Spatio-Temporal Expression of the Endogenous Locus in Embryonic Tissues

Overall levels of *Dlk1* expression were assessed in WT/WT and WT/TG embryonic tissues at E16 (Table S1) and E18 by TaqMan RT-qPCR for 70B (Figure 2A) and 70A (Table S1). Results show that *Dlk1* levels are over-expressed around 2–2.5 fold in embryonic tissues analysed in the WT/TG when compared to normal littermates (Figure 2A). The placenta was the only tissue with no significant over-expression (109%×±15% compared to WT/WT) (Figure 2A and Table S1). This suggests that key placenta specific regulators lie outside the region delineated by the transgene.

Western Blot analysis of DLK1 protein expression was conducted in control, WT/TG and TG/TG tissues and compared with the RT-qPCR analysis. In all cases, using this semi-quantitative method, protein levels correlated with RNA levels for the tissues and stages analysed (Figure 2B). Curiously, a slight increase in DLK1 protein levels was observed in the TG/TG placenta, suggesting that there is minimal expression of the transgene in this tissue.

**Figure 3:**
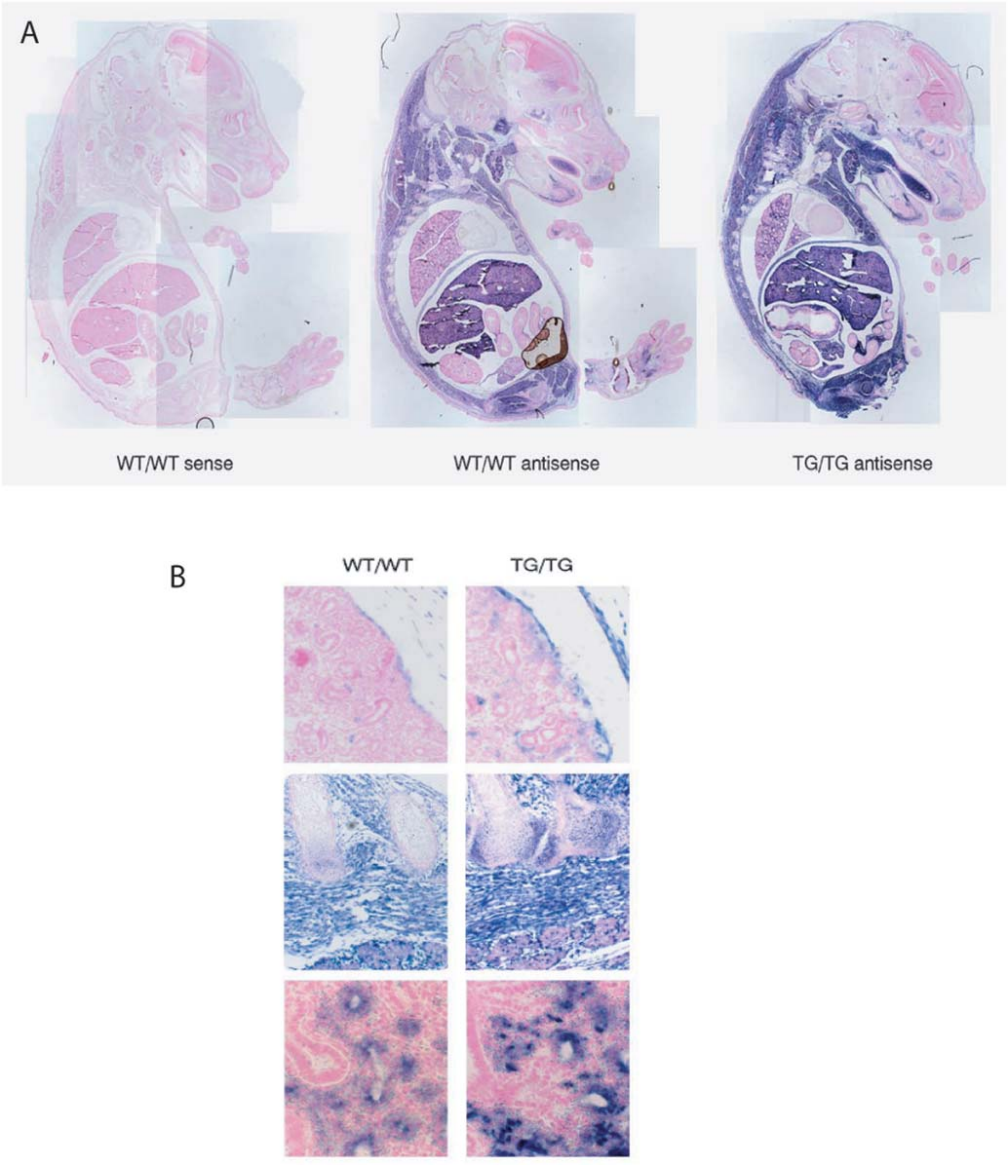
Transgenic expression in embryos recapitulates endogenous expression. A. WT/WT (left and middle) and TG/TG (right) e16 70B embryos were sectioned, then in-situ hybridisation was performed using sense control (left panel) and antisense probes to *Dlk1* (exons 3 to 5, middle and right panels). No ectopic expression was observed. 2.5× magnification. B. High power images of WT/WT (left) and TG/TG (right) kidney (20×, top), neck (10×) and lung (40×). In the WT/WT kidney, *Dlk1* is largely absent except in the capsule and some punctuate staining in the mesenchyme. This pattern is recapitulated in the TG/TG, with greater intensity. In the neck, *Dlk1* is expressed in the ossifying cartilage of the spinal cord, in the muscle and in the brown adipose tissue deposits, in both the WT/WT and TG/TG. In WT/WT lung *Dlk1* is expressed in the epithelium of branching alveoli and in the mesenchyme. This pattern is also clearly present in the TG/TG.

Tissue-specific expression of *Dlk1* was compared between normal and transgenic conceptuses by *in situ* hybridisation and no ectopic *Dlk1* activity was evident (Figure 3 and Table S4) at E16 or E18. This suggests that endogenous regulators are present on the transgene and are driving transgenic *Dlk1* transcription. Tissue-specific expression of the endogenous neighbouring non-coding RNA gene, *Gtl2* was unaffected in all genotypes (data not shown) allowing phenotypes of the transgenic mice to be attributed to *Dlk1* over-expression. Phenotypic characterization was performed independently in all three lines and all the observed phenotypes were consistent so data was combined. Results generated from the individual lines are presented and also summarised in the Supplementary Data.

### *Dlk1* Over-Dose Leads to Embryonic Growth Enhancement Independent of the Placenta

**Figure 4:**
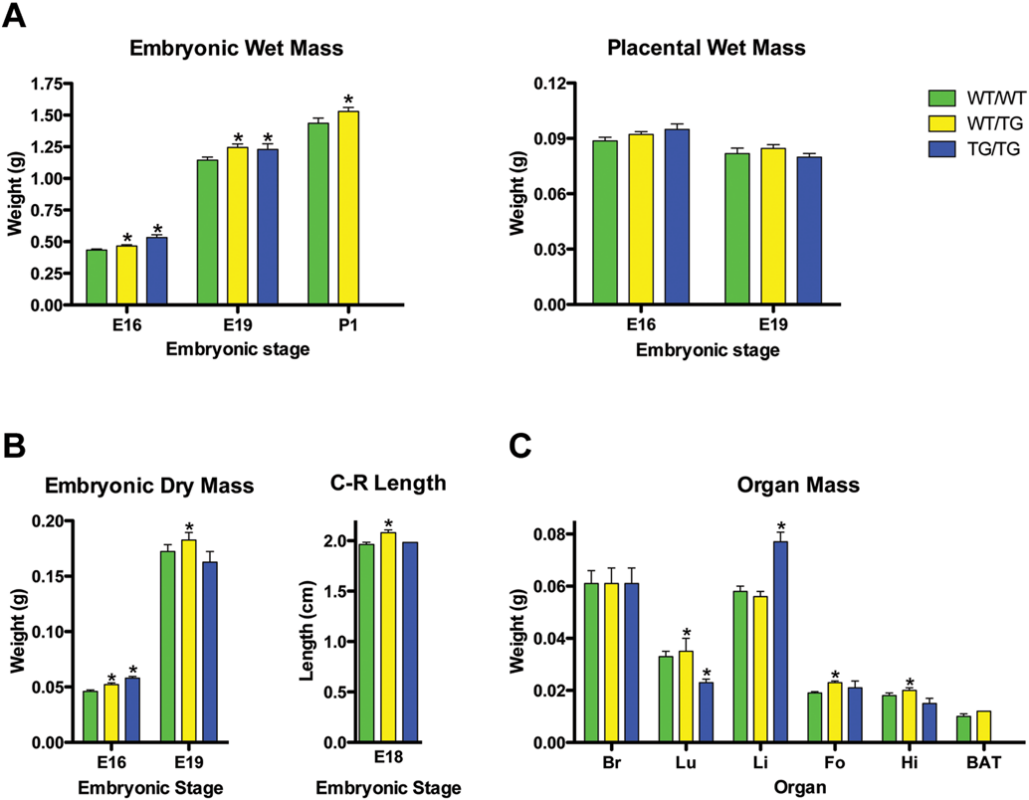
Dlk1 over-expression causes tissue-specific growth enhancement. (A) Embryonic and placental growth of wild-type and transgenic conceptuses and (B) embryonic dry masses and crown-rump lengths were measured from fetuses derived from heterozygous intercrosses (n≥5 litters; n≥9 animals per genotype). Graphs represent mean values for each genotype within a litter±SEM for multiple litters. Significant differences from wild-type are indicated by asterisks (p-value<0.05, paired, nonparametric Wilcoxon's test). (C) Wet mass of E18 brain (Br), lungs (Lu), liver (Li), forelimbs (Fo) and hindlimbs (Hi) and E19 brown adipose tissue (BAT) were measured in WT/WT, WT/TG and TG/TG fetuses (n≥3 litters; n≥10 animals per genotype, except TG/TG forelimbs and hindlimbs where n = 4). Graph represents mean weight±SEM per genotype. Significant differences from wild-type are indicated by asterisks on top of the WT/TG or TG/TG bar (p-value<0.05, unpaired Student's *t* test). Data shown in this figure are combined results from 70A, 70B and 70C.

Imprinted genes have long been known to function in controlling pre-natal growth and nutrient acquisition [20]. To address a growth function for *Dlk1*, we performed extensive measurements of wet and dry embryonic masses, placental wet masses and crown-rump lengths. WT/TG fetuses are expressing a double dose of *Dlk1* and showed consistent overgrowth from E16 to the day of birth. Wet and dry embryonic masses and crown-rump (C–R) length values increased by 6–10% compared to WT/WT littermates (Figure 4A–B; Table S3). TG/TG fetuses show growth enhancement at E16 (wet mass and dry mass increases of 23–25%). In contrast, no differences are observed in E18 C–R length and dry mass compared to WT/WT littermates most likely due to the failure to thrive of these severely compromised fetuses at the later stages as has been observed in other models [16]. Differences in wet mass between TG/TG and WT/WT at late gestation are due to oedema (Figure 6A–B). This late gestation data correlates with lethality of the TG/TG fetuses commencing around E16 (Table 1, Figure 4 and see below).

**Table 1:** Frequency and viability of *Dlk1* transgenic mice from E16 to early postnatal life.

TRANSMISSION	GENOTYPE	E16	E18–E19	P1–P3
**Maternal**	WT/WT	37	29	-
	TG/WT	48	47 (1)	-
**Paternal**	WT/WT	31	56	116 (12)
	WT/TG	45	41	133 (42)*
**Heterozygous Intercross**	WT/WT	34	58	13 (1)
	WT/TG	87 (1)	153	23 (4)
	TG/TG	37 (4)	80 (7)	7 (7)

The values represent number of animals genotyped in each time-point from crosses involving *Dlk1* transgenic animals; the numbers in brackets represent number of dead animals.

*: WT/TG neonatal lethality is statistically significantly increased compared to WT/WT (p-value<0.01; *X* ^2^-test).

**Figure 5:**
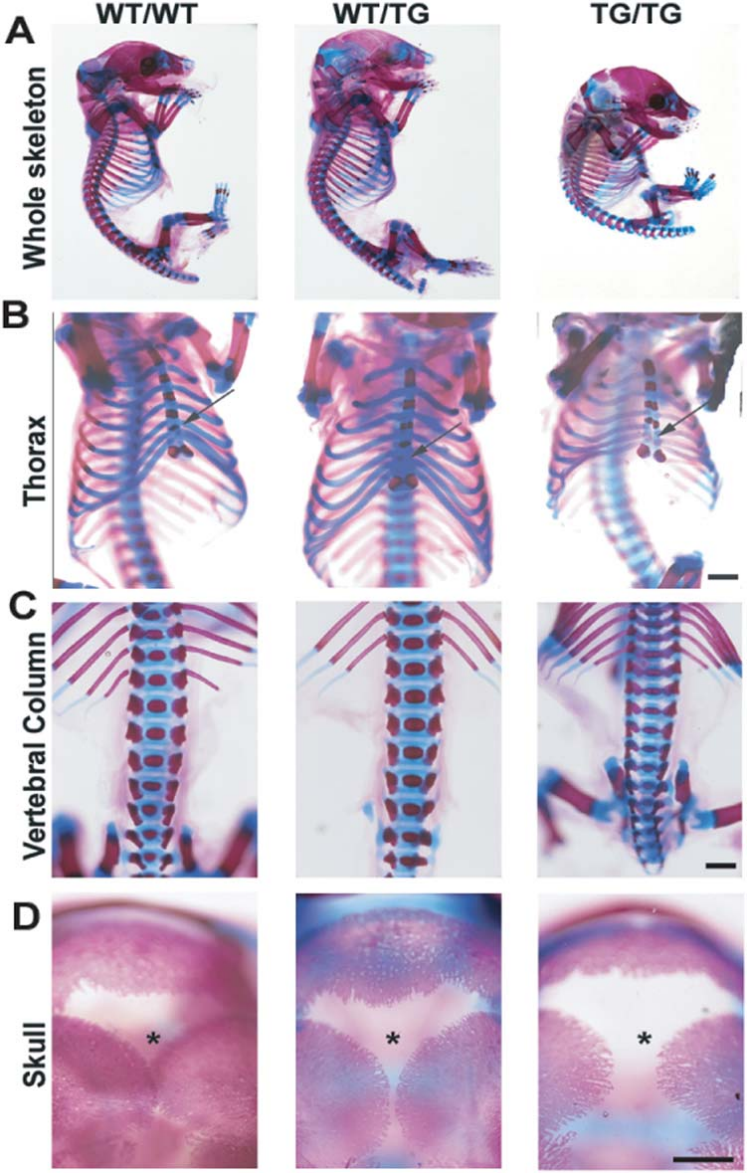
Skeletal defects in E19 WT/TG and TG/TG fetuses. A, Sagittal view of the whole skeleton. B, Frontal view of the thoracic cage. C, Dorsal view of the vertebrae in the dorsal zone and D, cranial view of the skull of E19 WT/WT, WT/TG and TG/TG skeletons stained with Alcian Blue/Alizarin Red. Arrow in B indicates the fifth ossification centre in the WT/WT sternum, absent in the WT/TG and TG/TG skeleton. Asterisk in D marks the closure of the sagittal suture. Scale bars: 0.5 mm (B and C) and 1 mm (D). All images shown A–D are derived from 70A animals; results are exactly the same for 70B and 70C.

**Figure 6:**
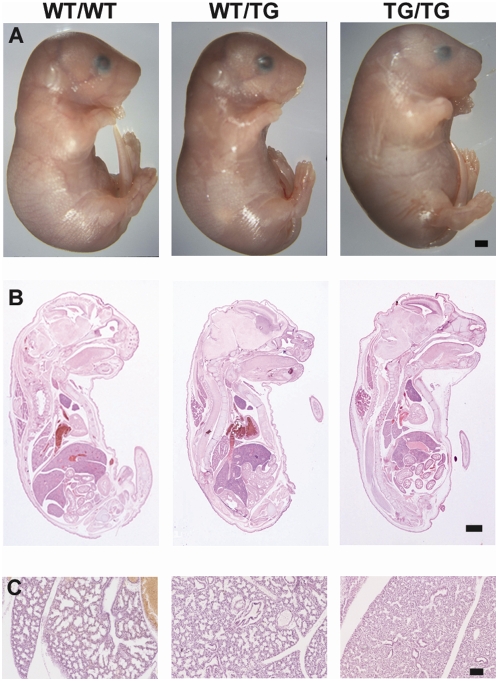
TG/TG fetuses exhibit major developmental abnormalities. A. E19 WT/WT WT/TG and TG/TG fetuses. B. Comparable mid-sagittal H&E sections through E18 WT/WT, WT/TG, and TG/TG fetuses. C. H&E stained sections through E18 lungs showing dense cellular arrangement in TG/TG lung. Sections shown are from 70A animals; results are the same for 70B and 70C. N±3 samples from each genotype were analysed for each line. Scale bar in A and B: 1 mm; Scale bar in C: 100 µm.

To determine the contribution of individual tissues to the increase in fetal mass, E18–19 brains, livers, lungs, forelimbs, hindlimbs and brown adipose tissue (BAT) were weighed and analysed histologically (Figure 4C, Figure 5, Figure 6). In the growth-enhanced WT/TG fetuses, lungs, forelimbs and hindlimbs were proportionally heavier. WT/TG brains, that significantly over-express *Dlk1*, were spared. E18 liver growth was unaffected, suggesting that the milder *Dlk1* over-expression in this tissue (E18: 1.31×±0.21 of WT/WT *Dlk1* liver expression and at E16: 1.67×±0.21) may not be sufficient to cause overgrowth. Overgrowth was observed in TG/TG livers evident from E16. Histological examination revealed no obvious defects and portal triads, and hepatocyte size appeared unaffected (Figure 4C, Figure 6 and data not shown). In general, this suggests that, with the exception of the brain, multi-organ overgrowth is associated with *Dlk1* over-expression in an organ-autonomous manner.

In the placentas, no significant differences between the genotypes were observed at any stage (Figure 4A and Table S3). Histological analyses indicated no obvious phenotypic abnormalities in the placenta at E16 and E19 for the three genotypes (data not shown). This is consistent with the finding that *Dlk1* is expressed at very low levels from the transgene in this tissue (Figure 2 and Table S1) and suggests that embryonic growth enhancement occurs independently of the placenta.

### *Dlk1* Dosage Modulates Skeletal Maturation

PatDi(12)/PatDp(dist12) and ΔIG-DMR^MAT^ exhibit costal cartilage defects and hypo-ossification of mesoderm-derived bones [16],[18],[19]. Since WT/TG embryos have increased crown-rump length, we examined the skeletal development of WT/TG and TG/TG mice. WT/TG skeletons show signs of growth enhancement and minor ossification delays in the sternum and the closure of the sagittal suture (Figure 5). Further increasing the dosage of *Dlk1* in TG/TG animals led to more severe skeletal defects. Overall the TG/TG skeleton was smaller and the delay in the closure of the sagittal suture was more pronounced. In addition, we observed a bell-shaped thorax with a hypo-ossified sternum and thinner ribs and vertebrae. We can therefore relate the severity of hypo-ossification with increasing levels of *Dlk1*.

Another major defect associated with PatDi(12)/PatDp(dist12) and ΔIG-DMR^MAT^ is skeletal muscle immaturity [16],[18],[19]. Interestingly, in contrast to the defects in the skeleton, no muscle maturation abnormalities were observed even when *Dlk1* dosage was tripled. Detailed morphometric analysis assessing myofiber diameter and % of myofibers with centrally located nuclei was performed on the E18 diaphragm and on a subset of muscles in the forearm and no differences were observed (Figure S3). *Dlk1* over-expression of all isoforms was confirmed at the protein level (Figure 2B). Therefore our data clearly show no prenatal muscle immaturity or hypertrophy induced by *Dlk1* over-expression from endogenous regulators.

### Increasing *Dlk1* Expression Reduces Embryonic and Perinatal Fitness in a Dose-Dependent Manner

Hemizygous transgenic embryos are viable and fertile and observed at Mendelian frequencies at birth indicating that a double dose of *Dlk1* expression is compatible with embryonic viability (Table 1 and. Table S2). In contrast to WT/TG animals, TG/TG embryos have distinct morphological features from E16 including severe oedema, a small thoracic region and a protruding abdomen (Figure 6A and B). This is associated with lethality from E16 with none of the TG/TG animals surviving more than a few hours after birth (Table 1 and Table S2). We also observed that the weight of the TG/TG lungs was significantly reduced (Figure 4C) with a denser cellular arrangement at E18 (Figure 6C). *Dlk1* is strongly expressed in the bronchioles of the lung (Figure 3 and ref [10]) and an intrinsic defect in lung development caused by over-expression of *Dlk1* is likely to contribute to the lethality of these animals.

Despite the absence of major embryonic abnormalities caused by doubling *Dlk1* dosage *in vivo*, we observed a significant increase in early postnatal lethality in WT/TG animals. 32% of WT/TG pups died during the first three days after birth compared to 10% of WT/WT littermates, in crosses from wild type mothers (Table 1 and Table S2). In order to understand the cause of death, we studied processes essential to early postnatal survival in rodents such as temperature regulation, suckling ability and glucose homeostasis.

**Figure 7:**
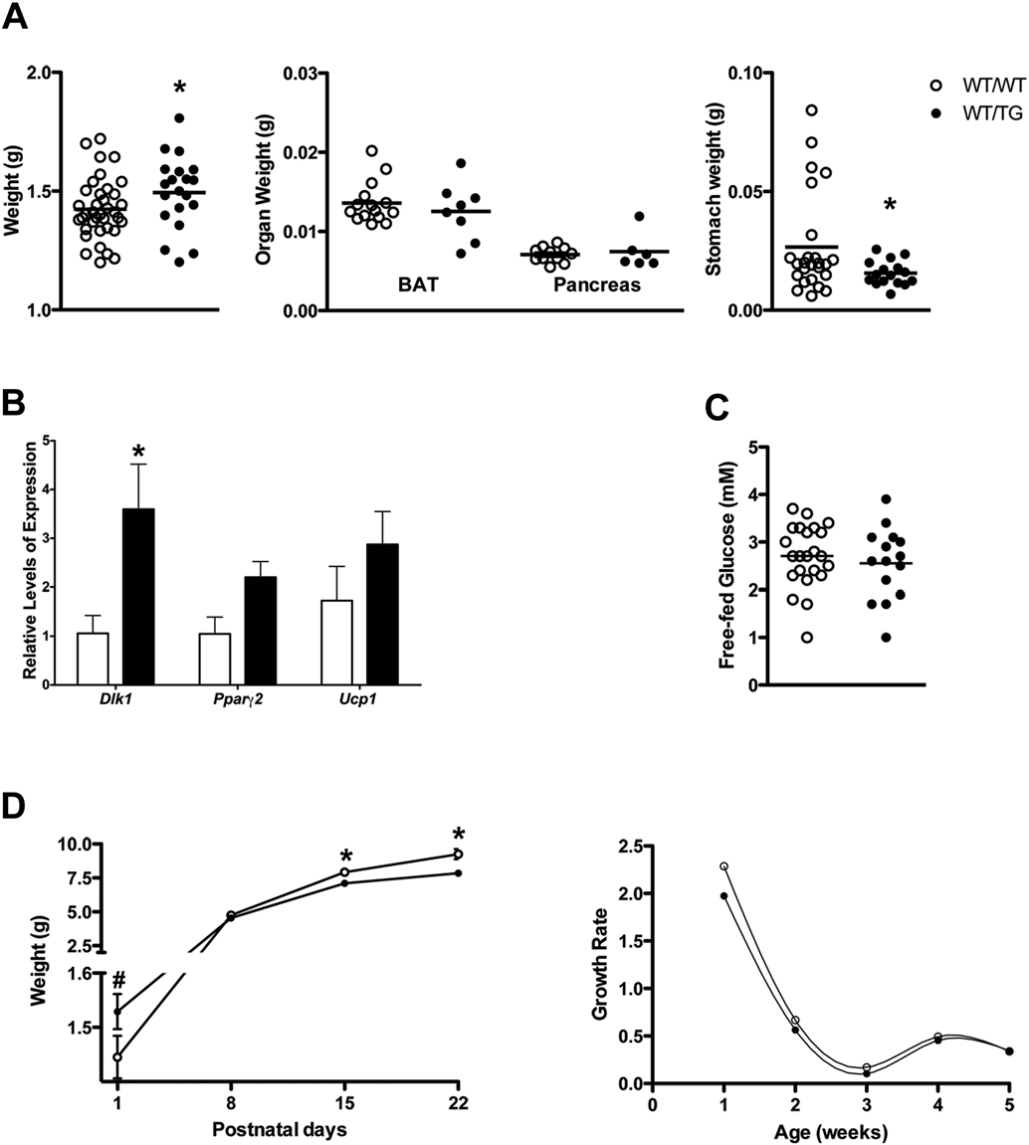
Poor early post-natal fitness in animals with *Dlk1* double dose. A. Neonatal total mass (left panel) and organ weights (right panels) at birth (P1). Graphs represent weight of individual animals/organs. Significant differences from wild-type are indicated by asterisks on top of the WT/TG (p-value<0.05, unpaired Student's *t* test). For stomach weights, the non-parametric Mann-Whitney U-test was performed (p-value<0.05). B. BAT expression analysis by TaqMan RT-qPCR. Graphic representation of relative levels of expression of *Dlk1*, *Pparγ2* and *Ucp1* normalized against 18S (loading control) at P1 (mean±SEM, n≥4). Significant differences between WT/TG and WT/WT for each gene are indicated by asterisks on top of the WT/TG bar (p-value<0.05; unpaired Student's *t* test). C. Free-fed glycemia at P1; Graph represents individual levels of glycemia (n≥15). D. The left panel represents the pre-weaning growth curve of WT/WT and WT/TG male juveniles. Graph represents mean values±SEM per genotype (n≥7). Significant reduced weight from WT/WT is indicated by # and significant increased weight from WT/WT is indicated by * (p-value<0.05, unpaired Student's *t* test). The right panel shows the growth rate of WT/WT and WT/TG animals in the first 5 weeks of age (calculated using the following formula: for week n: [weight at week n – weight at week (n-1)]/weight at week n. All data shown in A–D are the combined results for the three transgenic lines.

Temperature regulation is required for adapting to cold exposure after birth and relies on the process of non-shivering thermogenesis (NST) mediated by brown adipose tissue (BAT) metabolism. *Dlk1* is believed to inhibit adipogenesis of both brown and white adipose tissue [21],[22] thus is a likely candidate for involvement in the NST process. We measured BAT per body weight and analysed markers for both fat differentiation (such as *Pparγ2*) and thermogenesis (such as *Ucp1*) before and after birth. At E19, WT/TG BAT per body weight and levels of *Pparγ2* and *Ucp1* are comparable to WT/WT (Figure S4). At birth, we observed that *Ucp1* and *Pparγ2* expression was slightly elevated, perhaps to compensate for a slight decrease in BAT mass per body weight (Figure 7A and B). Failure to thrive, therefore, cannot be ascribed to compromised NST.

The suckling ability of *Dlk1* WT/TG pups at birth was assessed by using stomach weight as a measure of milk content [23]. We observed that WT/WT animals were frequently found with stomach content in the range of 0.03–0.08 grams, whilst WT/TG animals never exceeded 0.03 grams (Figure 7A). *In situ* data shows that at late gestation (E18), highest expression of *Dlk1* is in the tongue and the upper and lower lips, which is consistent with a role of *Dlk1* in suckling (Table S4 and data not shown). Blood glucose levels were not different at birth between the two genotypes (Figure 7C).

We also decided to monitor the growth performance of WT/TG animals during the first three weeks of life, when juveniles are still dependent on the mother for nutrition. WT/TG animals are born bigger than their littermates (Figure 4A and Figure 7A), but they fail to gain weight at the same rate as WT/WT neonates during the first two weeks (Figure 7D). By 14 days, WT/TG animals are small and remain small thereafter (∼10%) (Figure 7D and data not shown). The differences in growth performance are most pronounced within the first week after birth (Figure 7D), correlating with poor suckling and the increased lethality of these animals. In conclusion, despite an early growth advantage, animals expressing a double dose of *Dlk1* fail to thrive in early life, and thus any benefit conferred by an increased embryonic size is offset by postnatal lethality.

## Discussion

We have developed a unique model in which the consequences of a single, double and triple dosage of one imprinted gene, *Dlk1*, can be assessed in the growing embryo. The double dose is reminiscent of the situation where there is no imprinting of this gene. *BAC* and *YAC* transgenes have been widely used in mouse genetics and in imprinting studies as a molecular tool for the localisation of transcriptional or imprinting regulatory elements [24],[25],[26]. We generated transgenes differing in the extent of the sequences upstream of *Dlk1*. The Tg*^Dlk1^* ^-31^ did not express *Dlk1* in four transgenic lines, regardless of integration site or copy number (Figure S1A). In contrast, Tg*^Dlk1^* ^-70^, which extends from 49 kb upstream of the *Dlk1* gene, is expressed. We have therefore determined that the majority of embryonic tissue-specific enhancer sequences for *Dlk1* expression are located in the 8 kb to 49 kb interval upstream of the gene. This is consistent with a previous report showing that enhancers for *Dlk1* are absent from 3 kb upstream to 175 kb downstream of the gene [26]. Minimal transgene expression in the placenta suggests the absence of the specific regulatory sequences for this organ in the 70 kb transgene.

At E16, we detected growth enhancement in a *Dlk1* dose-dependent manner. TG/TG were more than 20% larger than WT/WT littermates, whereas WT/TG were growth-enhanced by 10%. At later stages, progressive morbidity and mortality of TG/TG embryos precluded a meaningful comparative assessment of their growth rate. This was not the case for viable WT/TG embryos which clearly maintained an increased growth trajectory from E16 up to birth. This is reciprocal to the 20% reduction in weight reported for *Dlk1*-null E19 fetuses [21]. In general, tissue-specific overgrowth correlated with levels of *Dlk1* over-expression, except in the brain. This suggests *Dlk1* acts locally on organ growth, though we cannot rule out an endocrine role [27].

With the exception of studies on placental specific *Igf2* [28],[29], previous functional analyses of imprinted genes expressed in embryo and placenta have not been able to discern whether growth effects are intrinsic to the embryo or are secondary to placental function. This is because genetic manipulation of imprinted genes often results in altered gene dosage in both embryonic and extra-embryonic tissues [reviewed in 30]. In contrast, the WT/TG model reported here generates embryonic *Dlk1* over-expression in the presence of a normal placenta with a normal *Dlk1* dose, and the results indicate that *Dlk1* can modulate embryonic growth independently of the placenta. To complement these studies, it will be important to determine the extent to which placental *Dlk1* expressed in the fetal endothelium and some trophoblast cells of the labyrinthine zone [10] also contributes to embryonic growth.

Importantly, this study shows that *Dlk1* has a dual role on growth. WT/TG neonates are born larger but fail to thrive during the first week of life. The reduced postnatal growth rate is concurrent with reduced food intake in the neonatal period. Maternally repressed imprinted genes are expected to favour growth performance and resource acquisition from embryonic stages to weaning according to the kinship theory which posits that imprinting arose as a consequence of a conflict between males and females over the allocation of maternal resources to the offspring [31],[32]. The embryonic overgrowth generated by *Dlk1* double dosage follows the directionality predicted by this theory however the postnatal failure to thrive phenotype is contrary to it. Our model implicates *Dlk1* in reduced postnatal nutrient acquisition. Indeed the results suggest that maternal repression of *Dlk1* may have evolved to increase postnatal nutrient acquisition. Furthermore it has been hypothesized that maternally repressed imprinted genes, such as *Dlk1*, would minimize heating contribution within huddles [33],[34]. NST is not impaired in BAT over-expressing *Dlk1*. In fact, slight over-expression of *Pparγ2* and *Ucp1* may suggest the opposite scenario, whereby a maternally repressed gene may contribute more to the communal heating. Our results are therefore inconsistent with the conflict hypothesis. It is possible that imprinting may have evolved due to more than one type of selective pressure, perhaps different for different domains.

In TG/TG embryos over-growth occurs at E16 coincident with an increased frequency of embryonic mortality. Embryos expressing this triple dose did not survive past birth. A double dose of *Dlk1* was compatible with embryonic viability but resulted in significant neonatal lethality. We propose a negative correlation between gene dosage and survival that may fix an upper limit on growth promotion by *Dlk1*.

One hypothesis for the lethality could be that *Dlk1* dosage regulates the balance between proliferation and differentiation resulting in a trade-off between pre-natal size and developmental maturity. Increasing *Dlk1* dosage incrementally shifts the embryo towards increased growth, perhaps at the expense of organ maturation as was suggested for PatDi(12) embryos [16]. The major abnormalities found in the liver, lungs and skeleton of the TG/TG fetuses may be signs of developmental immaturity of these organs. The inability of the lungs to support TG/TG animals surviving to term, as well as pre-natal oedema, is consistent with this. The *Notch* signalling pathway is required for branching morphogenesis and maturation of the lungs, and disruption of this pathway can lead to peri-natal lethality due to lung hypotrophy [reviewed in 35],[36],[37]. In contrast, except for subtle ossification delays in the WT/TG fetuses, no other clear signs of organ immaturity were identified, so we cannot conclude whether this contributes to the neonatal lethality of the hemizygous genotype.

In order to explore the cause of lethality in the WT/TG neonates more closely, we measured several parameters related to rodent well-being. WT/TG animals were significantly less likely to have milk-filled stomachs on the day of birth. This may be a result of defects in suckling behaviour engendered by many causes, such as appetite or olfactory regulation, or motor function. Starvation may therefore be the cause of death of WT/TG neonates, and reduced feeding in the first week is also a likely cause of the reduced growth rate of surviving transgenic animals during this period. Further analysis of the physiological consequences of this interesting growth trajectory of prenatal growth enhancement followed by postnatal compromised growth is in progress.

Imprinting of the *Dlk1-Dio3* cluster is important. Disruption of imprinted expression in several models results in lethality therefore there is a clear selective pressure to maintain imprinting at this cluster [13],[38]. The IG-DMR, like other imprinting control regions, controls the dosage of many linked genes. It has been postulated that in imprinted clusters, some genes are the target of dosage regulation while other are merely bystanders whose altered dosage do not have phenotypic consequences and therefore are not targets for selection [39]. We show that *Dlk1* is a dose-dependent regulator of embryonic growth, as well as modulating processes necessary for postnatal survival. WT/TG animals model loss of imprinting of *Dlk1*, and have significantly reduced fitness, suggesting that the maintenance of imprinting of solely *Dlk1* in eutherians by the IG-DMR has been sufficient to selectively retain imprinting control of the entire chromosome 12 cluster. However, the phenotypes of PatDi(12)/PatDp(dist12) and ΔIG-DMR^MAT^ embryos are much more severe than that of a *Dlk1* double-dose alone; therefore the dosage of at least one other gene in this cluster requires tight regulation. *Rtl1* is a likely candidate for this as defects have been reported in both knockout and overexpression models [38] and this gene is found only in eutherian mammals that have imprinting [12].

We cannot know the selective pressures surrounding the acquisition of imprinting at chromosome 12 in eutherians however this mechanism must have evolved to regulate dosage-critical genes. We have shown *Dlk1* to be such a dosage-critical gene through its significant consequences on animal fitness upon dosage modulation. It therefore could have been the target of selection for dosage control by imprinting at this locus. Current levels of *Dlk1* represent an optimal balance of maximized growth with minimal neonatal lethality.

## Materials and Methods

### Generation and Breeding of Transgenic Animals

Tg*^Dlk1^* ^-31^ and Tg*^Dlk1^* ^-70^ transgenes were obtained by restriction endonuclease mapping of the BAC clone 163O05, screened from a BAC library of the mouse strain 129/Sv. The two transgenes were microinjected individually into the male pronuclei of (C57BL/6×CBA) F1 zygotes and then implanted into foster (C57BL/6×CBA) F1 mothers. The first generation of animals was obtained by crossing the founder with a (C57BL/6×CBA) F1 animal. After the first generation, all transgenic animals were mated on a C57BL/6 background to maintain the lines. Animals were housed four per cage (maximum) in a temperature-controlled room (24°C) with a 12-hr light/dark cycle. Food and water were available *ad libitum*. All experiments involving mice were carried out in accordance with UK Government Home Office licensing procedures. For the embryonic studies, the day of vaginal plug was considered day E1.

### Expression Studies

RNA was extracted from whole embryos using TRI Reagent (Ambion), following the manufacturer's guidelines. mRNA was then isolated from 120 µg of total RNA using Dynalbeads Oligo (dT)_25_ kit (Dynal) following the supplied protocol. We carried out northern-blot hybridization and used probes as described previously for *Dlk1* and *Gtl2* and *Gapdh* [3]. For RT-qPCR, total RNA (10 µg) was DNase-treated with RQ1 RNase-free DNase (Promega) following the manufacturer's guidelines. All cDNA was synthesized using random hexamers and Superscript III RNase H^−^ Reverse Transcriptase (Invitrogen), following standard procedures.

Taqman quantitative real-time PCR (qRT-PCR) was used to measure expression levels of *Dlk1* and *Gtl2* normalized to *β-2-microglobulin* (*β2m*) in different E16 and E18 embryonic tissues. All RT-qPCR reactions were performed in a 25 µl final volume using standard Taqman qPCR conditions (Appplied Biosystems protocols) and amplified on a DNA engine Opticon 2 thermocycler (MJ Research). All reactions were conducted in triplicate. *Dlk1* expression levels were measured using the TaqMan gene expression assay ID - Mm00494477_m1 (Applied Biosystems). *Gtl2* gene expression was measured using the forward primer 5′-GGGCGCCCACAGAAGAA-3′, the reverse primer 5′- GGTGTGAGCCGATGATGTCA-3′ and the TaqMan MGB FAM probe FAM-5′-CTCTTACCTGGCTCTCT-3′-NFQ, spanning the *Gtl2* exon 1-exon 2 boundary. Finally, for the *β2m* gene, the forward primer B2M-32 5′-CACCCCCACTGAGACTGATACA-3′, the reverse primer B2M-38 5′- TGGGCTCGGCCATACTG-3′ and the TaqMan MGB VIC-labelled fluorogenic probe VIC-5′-CCTGCAGAGTTAAGC-3′- NFQ were used. Relative expression was calculated by normalisation to 100% using fetal E18 WT/WT tongue for the expression profile of the embryonic tissues and quantified using the comparative method (2^−dCt^) [40]. *Pparγ2* and *Ucp1* analysis was performed as previously described [41]. The fluorescent signal emitted during PCR was detected using the DNA engine Opticon 2 sequence detection system (MJ Research) and post-PCR data analysis was performed using the Opticon Monitor analysis software version 2.02 (MJ Research).

*In situ* hybridisation was conducted on paraformaldehyde fixed, wax embedded embryo and placenta sections at E16 and E18 of gestation according to the procedures previously described [42].

### DLK1 Protein Analysis

Tissue lysates from whole embryos, whole placentas and multiple organs were used for SDS/PAGE analysis with a 10% polyacrylamide gel. Resolved proteins were transferred to a poly-vinylidene difluoride (PVDF) Western blotting membranes Immobilon-P (Millipore) which were incubated with anti-DLK1 H-118 rabbit polyclonal antibody (1∶500) (Santa Cruz Biotechnology) or anti-DLK1 (1∶500) for placenta (Proteintech) and anti-α-TUBULIN mouse monoclonal antibody (Sigma) (1∶5000) overnight at 4°C, followed by the secondary HRP-conjugated antibodies: polyclonal goat anti-rabbit (1∶5000) or polyclonal goat anti-mouse (1∶7500) (DakoCytomotion), respectively, on the next day. Any signal was detected using ECL plus Western Detection System (Amersham Biosciences). The intensity of the signal was measured by scanning densiometry using Image J software (NIH).

### Phenotypic Characterisation of *Dlk1* Transgenic Fetuses and Neonates

All embryonic dissections were recorded in terms of number and genotype of embryos/pups, dead embryos/pups, necrotic embryos and reabsorptions for all *Dlk1* transgenic families. Wet masses were recorded for all fetuses/newborns/juveniles and placentas dissected at different developmental stages. Wet organ weights were also determined for E18, E19 and P1. For determination of the dry weight, pre-weighed embryos were dried by incubation at 60°C for 48 h plus 100°C for a further 24 h and, then, weighed. Crown-Rump length was accurately measured using callipers. Glycemia levels were measured using a One Touch Ultra glucometer (Lifescan).

For histology, freshly harvested embryos were fixed in 4% paraformaldehyde overnight at 4°C, dehydrated and embedded in paraffin wax using standard protocols. Sections of 7–10 µm were cut and 1 section in 10 was stained with Haematoxylin and Eosin (H&E). For skeletal preparation, the skeleton of E19 embryos was stained with Alcian Blue and Alizarin Red as described previously [16]. Muscle was analysed after immunocytochemistry with MY32 antibody (M4276 Sigma), a monoclonal antibody specific for skeletal muscle myosin heavy chain, performed according to the procedure described previously [16]. Comparative morphometrics were carried out on MY32-stained histological sections through the largest cross-sectional area of the forelimbs and diaphragm. Comparable sections were carefully selected for the TG/TG, WT/TG and WT/WT littermates and 3 sections (7 µm thick) with 20 section intervals were selected for each embryo. Measurements were performed by randomly selecting the fields across the whole section. Details were as described previously [19].

### Ethics statement

All animal work in this study was conducted under a licence (80/2042) from the UK Government Home Office.

## Supporting Information

Figure S1 *Dlk1* transgene copy number and expression. A. Schematic representation of the endogenous and transgenic Dlk1 locus; Restriction digestion with *Eco*RI (arrows) results in a 4.5 kb band for the endogenous gene and a 4.0 kb band for the transgenic copy, when detected by the probe represented in green. B. Determination of copy number in transgenic and normal (N) animals from different families (upper panel). Genotyping of the WT/TG and TG/TG animals of the 70C and 70A families (lower panels).
(2.47 MB TIF)

Figure S2 Tg^*Dlk1*-70^ is expressed at at double dose in all three lines and is not imprinted. A. Sequence analysis of RT–PCR products from E16 WT/WT, TG/WT (maternal transmission) and WT/TG (paternal transmission) embryos showing expression of the transgene regardless of parental-origin; the endogenous locus is indicated by the T allele (DBA/2) in the nucleotide marked by the arrow head, while the transgene is indicated by the C allele (129/Sv). Allele-specific sequence analysis was conducted for 70B and 70C families. B. Quantitative Northern blotting and histograms showing that *Dlk1* is expressed at twice the normal dose in WT/TG animals in all three transgenic lines analysed.
(3.57 MB TIF)

Figure S3 Transgenic mice do not have skeletal muscle defects. A. Comparable sections through the diaphragm of E18 PatDi(12), WT/WT, WT/TG and TG/TG fetuses stained with the myofibril specific antibody MY32. Scale bar: 50 µm. B. Morphometric analysis of the skeletal muscle of E18 WT/WT, WT/TG and TG/TG fetuses (a) Standard section through the forearm stained with MY32 antibody; Forel I (extensor carpi radialis longus+brachioradialis), Forel II (extensor digitorum+extensor carpi ulnaris) were used for morphometric measurements; Scale bar: 200 µm; Abbreviations: r -radius; u -ulna. (b) Morphometric analysis of the myofiber diameter of the skeletal muscles (diaphragm, forel I and forel II) of E18 WT/WT, WT/TG and TG/TG. Graphs show mean values±SEM (n≥4). (c) Morphometric analysis of the percentage of myofibers with centrally-located nuclei from the same material used to analyse myofiber diameter; Graphs show mean values±SEM (n≥4).
(2.32 MB TIF)

Figure S4 BAT expression analysis by TaqMan RT-qPCR at E19. Graphic representation of relative levels of expression of *Pparγ2* and *Ucp1* normalized against 18S (loading control) at E19 (mean±SEM, n≥6) (70B).
(0.43 MB TIF)

Table S1 Summary of the copy number and *Dlk1* expression levels in E16 WT/TG fetuses relative to wild type for the 70 kb *Dlk1* transgenic lines. Significant differences in expression for the different families are indicated by asterisks (p-value<0.05; unpaired student's *t*-test); Abbreviations: F3 - Generation 3.
(0.05 MB DOC)

Table S2 Frequency and viability of WT/WT, WT/TG and TG/TG animals from E16 to early postnatal life for the three over-expressing 70 kb transgenic lines (70A, 70B and 70C). The values represent number of animals genotyped in each time-point from crosses involving *Dlk1* transgenic animals; the numbers in brackets represent number of dead animals.
(0.06 MB DOC)

Table S3 Overall embryonic and placental growth of *Dlk1* transgenic fetuses from the three over-expressing 70 kb transgenic lines (70B, 70A and 70C). Values represent mean±SEM; Means were obtained from fetuses originated from at least three independent heterozygous intercross litters (n≥5). Percentage values represent the ratio between WT/TG or TG/TG weights to the WT/WT counterparts. Significant differences between the WT/TG and WT/WT and between TG/TG and WT/WT are indicated by asterisks on the percentage value (unpaired Student's *t* test).
(0.07 MB DOC)

Table S4 Comparison of expression of *Dlk1* between WT/WT and WT/TG or TG/TG embryos. In situ hybridisation was conducted on E16 and E18 conceptuses using antisense and sense (control) probes for *Dlk1*. Sections were scored for expression of *Dlk1*. Expression of *Dlk1* was evident in all endogenous sites and ectopic expression was not observed in transgenic embryos or placentas.
(0.04 MB DOC)
