# Supplementary figures and images for: Gene Dosage Effects of the Imprinted Delta-Like Homologue 1 (Dlk1/Pref1) in Development: Implications for the Evolution of Imprinting
Source: PLoS Genet. 2009 Feb 27;5(2):e1000392. doi: 10.1371/journal.pgen.1000392 (PMC2640098; doi:10.1371/journal.pgen.1000392)

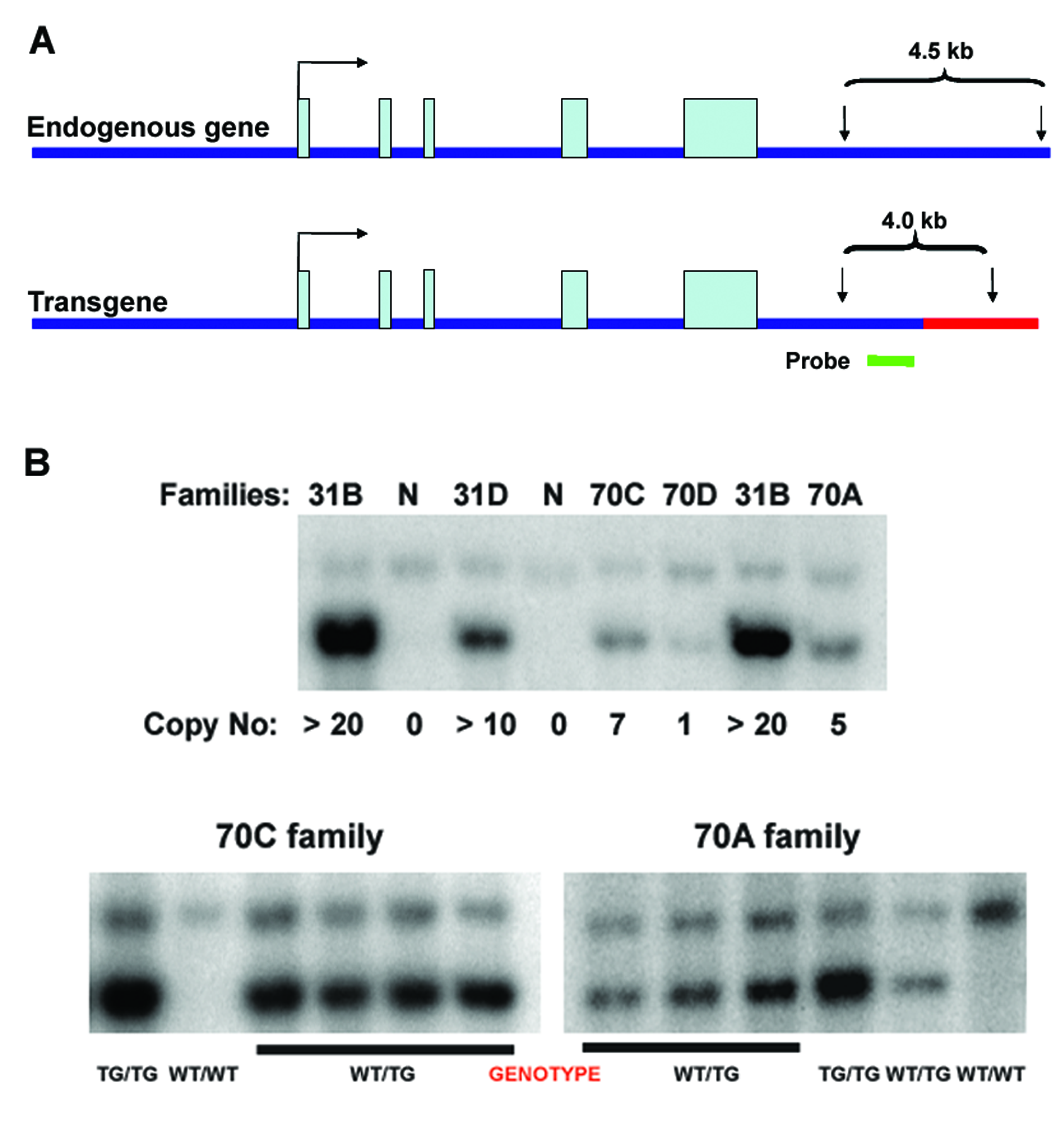

Supplement: Figure S1 — Dlk1 transgene copy number and expression. A. Schematic representation of the endogenous and transgenic Dlk1 locus; Restriction digestion with EcoRI (arrows) results in a 4.5 kb band for the endogenous gene and a 4.0 kb band for the transgenic copy, when detected by the probe represented in green. B. Determination of copy number in transgenic and normal (N) animals from different families (upper panel). Genotyping of the WT/TG and TG/TG animals of the 70C and 70A families (lower panels). (2.47 MB TIF) [file pgen.1000392.s001.tif]

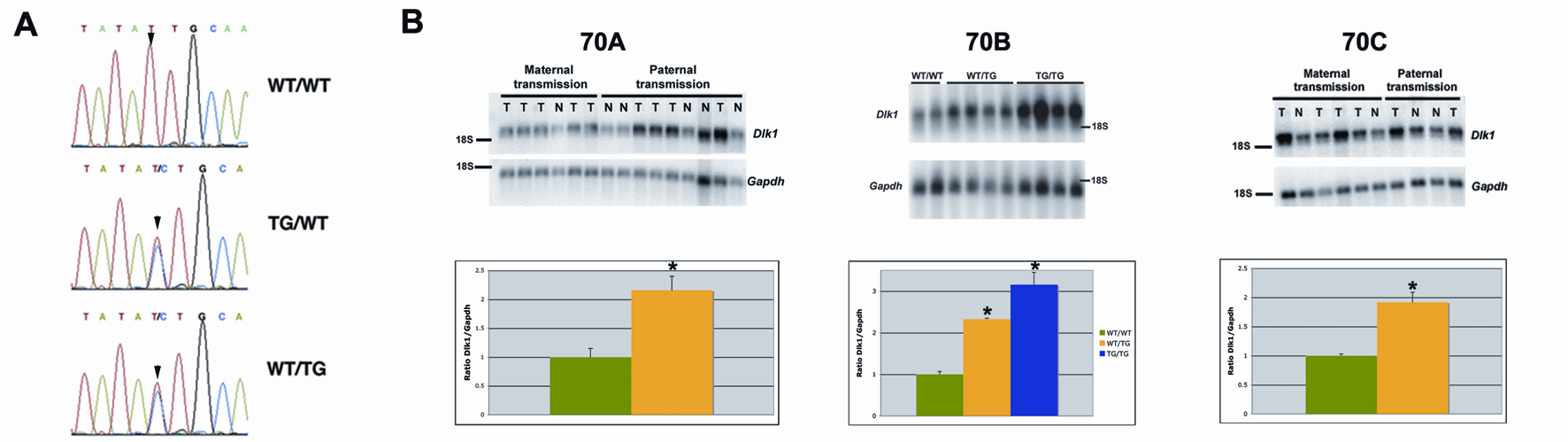

Supplement: Figure S2 — TgDlk1-70 is expressed at at double dose in all three lines and is not imprinted. A. Sequence analysis of RT–PCR products from E16 WT/WT, TG/WT (maternal transmission) and WT/TG (paternal transmission) embryos showing expression of the transgene regardless of parental-origin; the endogenous locus is indicated by the T allele (DBA/2) in the nucleotide marked by the arrow head, while the transgene is indicated by the C allele (129/Sv). Allele-specific sequence analysis was conducted for 70B and 70C families. B. Quantitative Northern blotting and histograms showing that Dlk1 is expressed at twice the normal dose in WT/TG animals in all three transgenic lines analysed. (3.57 MB TIF) [file pgen.1000392.s002.tif]

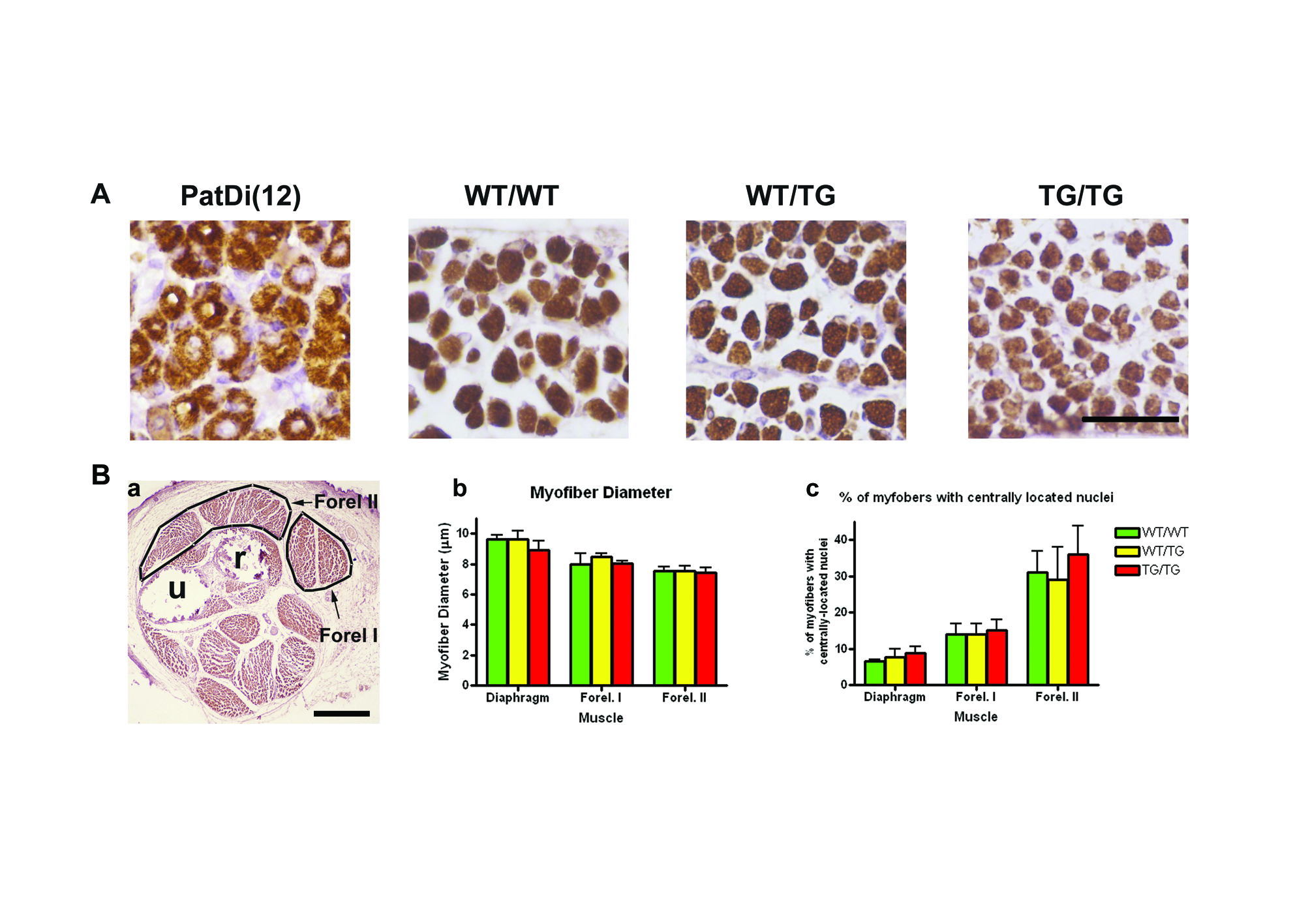

Supplement: Figure S3 — Transgenic mice do not have skeletal muscle defects. A. Comparable sections through the diaphragm of E18 PatDi(12), WT/WT, WT/TG and TG/TG fetuses stained with the myofibril specific antibody MY32. Scale bar: 50 µm. B. Morphometric analysis of the skeletal muscle of E18 WT/WT, WT/TG and TG/TG fetuses (a) Standard section through the forearm stained with MY32 antibody; Forel I (extensor carpi radialis longus+brachioradialis), Forel II (extensor digitorum+extensor carpi ulnaris) were used for morphometric measurements; Scale bar: 200 µm; Abbreviations: r -radius; u -ulna. (b) Morphometric analysis of the myofiber diameter of the skeletal muscles (diaphragm, forel I and forel II) of E18 WT/WT, WT/TG and TG/TG. Graphs show mean values±SEM (n≥4). (c) Morphometric analysis of the percentage of myofibers with centrally-located nuclei from the same material used to analyse myofiber diameter; Graphs show mean values±SEM (n≥4). (2.32 MB TIF) [file pgen.1000392.s003.tif]

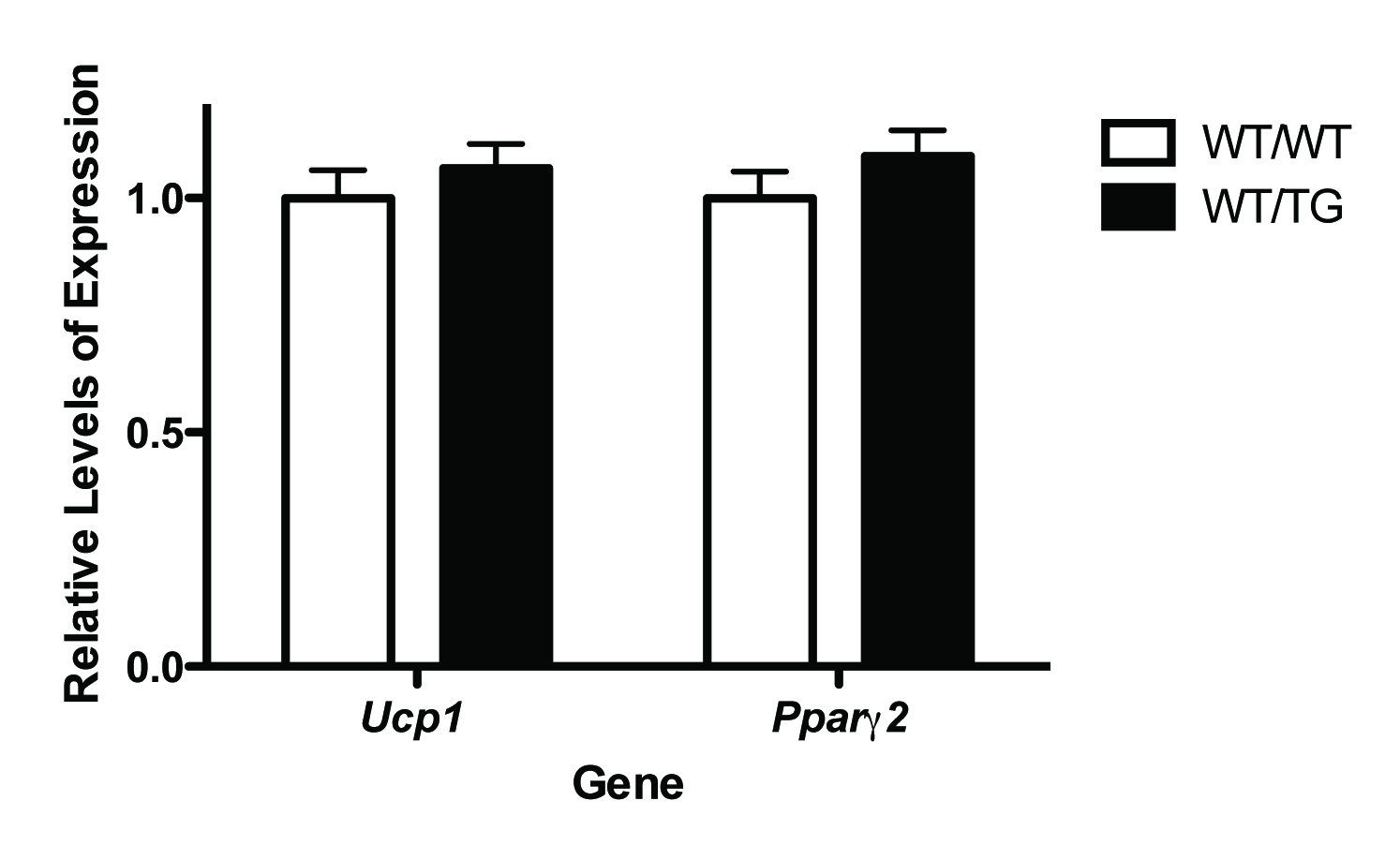

Supplement: Figure S4 — BAT expression analysis by TaqMan RT-qPCR at E19. Graphic representation of relative levels of expression of Pparγ2 and Ucp1 normalized against 18S (loading control) at E19 (mean±SEM, n≥6) (70B). (0.43 MB TIF) [file pgen.1000392.s004.tif]
