# Supplementary material for: Gene Dosage Effects of the Imprinted Delta-Like Homologue 1 (Dlk1/Pref1) in Development: Implications for the Evolution of Imprinting
Source: PLoS Genet. 2009 Feb 27;5(2):e1000392. doi: 10.1371/journal.pgen.1000392 (PMC2640098; doi:10.1371/journal.pgen.1000392)
Supplement: Table S1 — Summary of the copy number and Dlk1 expression levels in E16 WT/TG fetuses relative to wild type for the 70 kb Dlk1 transgenic lines. Significant differences in expression for the different families are indicated by asterisks (p-value<0.05; unpaired student's t-test); Abbreviations: F3 - Generation 3. (0.05 MB DOC) [file pgen.1000392.s005.doc]

**Table S1: Summary of the expression profile and phenotype of the three expressing 70 kb *Dlk1* transgenic lines**

|  | **70A** | **70B** | **70C** |
| --- | --- | --- | --- |
| **Copy Number** | 4-5 | 5-6 | 7 |
| ***Dlk1* whole embryo expression**  **(Northern blot)^a^** | WT/WT: 1.00x ± 0.15 (n=6)  WT/TG: 2.16x ± 0.24 (n=9)* | WT/WT: 1.00x ± 0.07 (n=3)  WT/TG: 2.33x ± 0.02 (n=4)*  TG/TG: 3.16x ± 0.29 (n=4)* | WT/WT: 1.00x ± 0.03 (n=4)  WT/TG: 2.16x ± 0.24 (n=6)* |
| ***Dlk1* whole embryo expression**  **(RT-qPCR) ^a^** | E18  WT/WT: 1.00x ± 0.05 (n=3)  WT/TG: 1.73x ± 0.46 (n=7) P=0.06  TG/TG: 2.74x ± 0.44 (n=6)* | E16  WT/WT: 1.00x ± 0.27 (n=7)  WT/TG: 2.32x ± 0.18 (n=14)*  TG/TG: 3.53x ± 0.12 (n=4)*  E18  WT/WT: 1.00x ± 0.41 (n=3)  WT/TG: 2.05x ± 0.34 (n=3)* | E16  WT/WT: 1.00x ± 0.23 (n=3)  WT/TG: 2.10x ± 0.25 (n=6)*  TG/TG: 2.90x ± 0.70 (n=3)* |
| **Transgene imprinting status** | No imprinting | No imprinting | No imprinting |
| ***Dlk1* placenta expression (RT-qPCR)^a^** | E18  WT/WT: 1.00x ± 0.20 (n=3)  WT/TG: 1.30x ± 0.46 (n=7) | E16  WT/WT: 1.00 ± 0.02 (n=3)  WT/TG: 1.16 ± 0.15 (n=8)  TG/TG: 1.27 ± 0.14 (n=5) P=0.08  E18  WT/WT: 1.00 ± 0.08 (n=6)  WT/TG: 1.09 ± 0.15 (n=6)  TG/TG: 1.42 ± 0.44 (n=5) | E18  WT/WT: 1.00x ± 0.20 (n=3)  WT/TG: 0.95x ± 0.40 (n=3)  TG/TG: 1.48x ± 0.52 (n=3) |
| **Tissue-specific expression (RT-qPCR)^a^** | E18 (n =3)  LUNG  WT/WT: 1.00x ± 0.28  WT/TG: 2.20x ± 0.51*  LIVER  WT/WT: 1.00x ± 0.41  WT/TG: 1.80x ± 0.65  TONGUE  WT/WT: 1.00X ± 0.27  WT/TG: 2.32X ± 0.39*    FORELIMBS  WT/WT: 1.00X ± 0.35  WT/TG: 2.50X ± 0.58* | E16 (n ≥ 3)  BRAIN  WT/WT: 1.00x ± 0.28  WT/TG: 2.21x ± 0.04*  LIVER  WT/WT: 1.00x ± 0.07  WT/TG: 1.67x ± 0.36*  HINDLIMBS  WT/WT: 1.00x ± 0.53  WT/TG: 1.80x ± 1.18    E18 (n ≥ 5)  BRAIN  WT/WT: 1.00x ± 0.07  WT/TG: 3.21x ± 0.37*  HEART:  WT/WT: 1.00x ± 0.12  WT/TG: 2.55x ± 0.35*  LUNG:  WT/WT: 1.00x ± 0.11  WT/TG: 1.61x ± 0.20*  LIVER:  WT/WT1.00x ± 0.10  WT/TG: 1.31x ± 0.21  PANCREAS  WT/WT: 1.00x ± 0.16  WT/TG: 1.65x ± 0.14*    TONGUE  WT/WT: 1.00x ± 0.07  WT/TG: 1.87x ± 0.11*  HINDLIMBS:  WT/WT: 1.00x ± 0.17  WT/TG: 2.99x ± 0.40  BAT:  WT/WT: 1.00x ± 0.06  WT/TG: 2.41x ± 0.44*  KIDNEY:  WT/WT: 1.00x ± 0.10  WT/TG 2.49x ± 0.21* | ND |
| Embryonic growth (E16, E19)^b^ | WT/TG: Overgrowth (E19)  TG/TG: Overgrowth (E19) | WT/TG: Overgrowth (E16, E19)  TG/TG: Overgrowth (E16 only) | WT/TG: Overgrowth (E19)  TG/TG: Overgrowth (E16, E19) |
| Placenta growth and histology (E16 and E19) ^b^ | Unaffected | Unaffected | Unaffected |
| Skeletal maturation | WT/TG: Minor ossification delays  TG/TG: Severe skeletal defects | WT/TG: Minor ossification delays  TG/TG: Severe skeletal defects | WT/TG: Minor ossification delays  TG/TG: Severe skeletal defects |
| Skeletal muscle maturation | Normal | Normal | ND |
| Lethality^c^ | WT/TG: Increased early post-natal lethality  TG/TG: Late gestation & perinatal lethality | WT/TG: Increased early post-natal lethality  TG/TG: Late gestation & perinatal lethality | WT/TG: Increased early post-natal lethality  TG/TG: Late gestation & perinatal lethality |

^a^ Expression profile has been normalised to 100% expression for the WT/WT counterpart for each tissue type in each line

Significant differences in expression compared to WT/WT counterpart are indicated by asterisks (p-value < 0.05; unpaired student’s t-test);

b This data is displayed in detail in Table S3;

c This data is displayed in detail in Table S2.
