# Supplementary material for: Gene Dosage Effects of the Imprinted Delta-Like Homologue 1 (Dlk1/Pref1) in Development: Implications for the Evolution of Imprinting
Source: PLoS Genet. 2009 Feb 27;5(2):e1000392. doi: 10.1371/journal.pgen.1000392 (PMC2640098; doi:10.1371/journal.pgen.1000392)
Supplement: Table S2 — Frequency and viability of WT/WT, WT/TG and TG/TG animals from E16 to early postnatal life for the three over-expressing 70 kb transgenic lines (70A, 70B and 70C). The values represent number of animals genotyped in each time-point from crosses involving Dlk1 transgenic animals; the numbers in brackets represent number of dead animals. (0.06 MB DOC) [file pgen.1000392.s006.doc]

**Table S2:**

| **70A** | **Genotype** | **E16** | **E18-E19** | **P1-P3** |
| --- | --- | --- | --- | --- |
| **Maternal Transmission** | WT/WT | 1 | 13 | - |
|  | TG/WT | 5 | 11 (1) | - |
| **Paternal Transmission** | WT/WT | 4 | 2 | 57 (4) |
|  | WT/TG | 4 | 7 | 61 (24) |
| **Heterozygous Intercross** | WT/WT | 3 | 13 | 5 (1) |
|  | WT/TG | 23 | 38 | 9 (2) |
|  | TG/TG | 6 | 20 (3) | 1 (1) |

| **70B** | **Genotype** | **E16** | **E18-E19** | **P1-P3** |
| --- | --- | --- | --- | --- |
| **Maternal Transmission** | WT/WT | 31 | 8 | - |
|  | TG/WT | 38 | 18 | - |
| **Paternal Transmission** | WT/WT | 19 | 35 | 37 (4) |
|  | WT/TG | 35 | 22 | 43 (9) |
| **Heterozygous Intercross** | WT/WT | 15 | 28 | 3 |
|  | WT/TG | 54 (1) | 74 | 6 |
|  | TG/TG | 24 (3) | 45 (1) | 5 (5) |

| **70C** | **Genotype** | **E16** | **E18-E19** | **P1-P3** |
| --- | --- | --- | --- | --- |
| **Maternal Transmission** | WT/WT | 5 | 8 | - |
|  | TG/WT | 5 | 18 | - |
| **Paternal Transmission** | WT/WT | 8 | 19 | 22 (4) |
|  | WT/TG | 6 | 12 | 29 (9) |
| **Heterozygous Intercross** | WT/WT | 6 | 17 | 5 |
|  | WT/TG | 10 | 41 | 8 (2) |
|  | TG/TG | 7 (1) | 15 (3) | 1 (1) |
