# Supplementary material for: Gene Dosage Effects of the Imprinted Delta-Like Homologue 1 (Dlk1/Pref1) in Development: Implications for the Evolution of Imprinting
Source: PLoS Genet. 2009 Feb 27;5(2):e1000392. doi: 10.1371/journal.pgen.1000392 (PMC2640098; doi:10.1371/journal.pgen.1000392)
Supplement: Table S3 — Overall embryonic and placental growth of Dlk1 transgenic fetuses from the three over-expressing 70 kb transgenic lines (70B, 70A and 70C). Values represent mean±SEM; Means were obtained from fetuses originated from at least three independent heterozygous intercross litters (n≥5). Percentage values represent the ratio between WT/TG or TG/TG weights to the WT/WT counterparts. Significant differences between the WT/TG and WT/WT and between TG/TG and WT/WT are indicated by asterisks on the percentage value (unpaired Student's t test). (0.07 MB DOC) [file pgen.1000392.s007.doc]

**Table S**3:

| **LINE** | **Embryonic age** | **Genotype** | **Embryonic wet mass (g)** | **Placental wet mass (g)** | | **Embryonic dry mass (g)** |
| --- | --- | --- | --- | --- | --- | --- |
| **70B** | **E14** | **WT/WT** | 0.140 ± 0.005 | 0.078 ± 0.004 | | 0.014 ± 0.001 |
|  |  | **WT/TG** | 0.147 ± 0.003  **105%** | 0.079 ±0.003  **101%** | | 0.014 ± 0.000  **100%** |
|  |  | **TG/TG** | 0.144 ± 0.004  **103%** | 0.078 ± 0.004  **100%** | | 0.014 ± 0.000  **100%** |
|  | **E16** | **WT/WT** | 0.430 ± 0.011 | 0.089 ± 0.001 | | 0.046 ± 0.001 |
|  |  | **WT/TG** | 0.470 ± 0.011  **109%*** | 0.091 ± 0.001  **102%** | | 0.052 ± 0.001  **113%*** |
|  |  | **TG/TG** | 0.558 ± 0.024  **130%*** | 0.096 ± 0.004  **108%** | | 0.058 ± 0.001  **126%*** |
|  | **E19** | **WT/WT** | 1.192 ± 0.015 | 0.078 ± 0.004 | | 0.182 ± 0.010 |
|  |  | **WT/TG** | 1.296 ± 0.012  **109%*** | 0.084 ± 0.001  **108%** | | 0.191 ± 0.013  **105%*** |
|  |  | **TG/TG** | 1.313 ± 0.034  110% | 0.081 ± 0.003  104% | | 0.161 ± 0.015  88% |
|  | **P1** | **WT/WT** | 1.445 ± 0.039 | na | | nd |
|  |  | **WT/TG** | 1.529 ± 0.032  **106*** | na | | nd |
|  | | | | | | |
| **70A** | **E16** | **WT/WT** | 0.429 ± 0.030 | 0.088 ± 0.004 | nd | |
|  |  | **WT/TG** | 0.419 ± 0.008  **98%** | 0.088 ± 0.002  **100%** | nd | |
|  |  | **TG/TG** | 0.481 ± 0.038  **115%** | 0.094 ± 0.002  **107%** | nd | |
|  | **E19** | **WT/WT** | 1.126 ± 0.020 | 0.080 ± 0.004 | 0.146 ± 0.005 | |
|  |  | **WT/TG** | 1.230 ± 0.026  **109%*** | 0.080 ± 0.003  **100%** | 0.168 ± 0.006  **115%*** | |
|  |  | **TG/TG** | 1.238 ± 0.028  **110%*** | 0.075 ± 0.002  **94%** | nd | |
|  | | | | | | |
| **70C** | **E16** | **WT/WT** | 0.454 ± 0.012 | 0.088 ± 0.003 | nd | |
|  |  | **WT/TG** | 0.455 ± 0.013  **100%** | 0.094 ± 0.003  **107%** | nd | |
|  |  | **TG/TG** | 0.501 ± 0.015  **110%*** | 0.088 ± 0.004  **100%** | nd | |
|  | **E19** | **WT/WT** | 1.006 ± 0.047 | 0.087 ± 0.005 | 0.164 ± 0.002 | |
|  |  | **WT/TG** | 1.256 ± 0.029  **125%*** | 0.083 ± 0.002  **95%** | 0.180 ± 0.003  **110%*** | |
|  |  | **TG/TG** | 1.288 ± 0.044  **128%*** | 0.086 ± 0.001  **99%** | 0.178 ± 0.010  **108%** | |
