# Supplementary material for: Gene Dosage Effects of the Imprinted Delta-Like Homologue 1 (Dlk1/Pref1) in Development: Implications for the Evolution of Imprinting
Source: PLoS Genet. 2009 Feb 27;5(2):e1000392. doi: 10.1371/journal.pgen.1000392 (PMC2640098; doi:10.1371/journal.pgen.1000392)
Supplement: Table S4 — Comparison of expression of Dlk1 between WT/WT and WT/TG or TG/TG embryos. In situ hybridisation was conducted on E16 and E18 conceptuses using antisense and sense (control) probes for Dlk1. Sections were scored for expression of Dlk1. Expression of Dlk1 was evident in all endogenous sites and ectopic expression was not observed in transgenic embryos or placentas. (0.04 MB DOC) [file pgen.1000392.s008.doc]

**Table S4:**

| **Tissue/Organ** | **WT/WT** | **WT/TG and TG/TG** |
| --- | --- | --- |
| Midbrain, Thalamus and Medula Oblongata | - | - |
| Hypothalamus | √ | √ |
| Pituitary | √ | √ |
| Upper and Lower Lips | √ | √ |
| Tongue | √ | √ |
| Salivary Glands | √ | √ |
| Bronchioles epithelium | √ | √ |
| Lung mesenchyme | - | - |
| Heart | - | - |
| Liver (majority) | - | - |
| Liver (individual hepatocytes) | √ | √ |
| Pancreatic stroma | √ (very weak) | √ (very weak) |
| Pancreatic acini | - | - |
| Stomach | - | - |
| Intestine | - | - |
| Kidney | - | - |
| Brown adipose tissue | √ | √ |
| Spinal Cord | - | - |
| Skeletal muscles | √ | √ |
| Mesenchyme | √ | √ |
| Growing cartilage | √ | √ |
| Hypertrophic cartilage | - | - |
| Placenta: Labyrinth zone (endothelial cells) | √ | √ |
| Placenta: Junctional zone and Decidua basalis | - | - |
